# New monocyclic monoterpenoid glycoside from *Mentha haplocalyx* Briq.

**DOI:** 10.1186/1752-153X-6-37

**Published:** 2012-05-06

**Authors:** Gai-Mei She, Chao Xu, Bin Liu

**Affiliations:** 1 School of Chinese Pharmacy, Beijing University of Chinese Medicine, Beijing 100102, P. R. China

## Abstract

Two new monocyclic monoterpenoid glycosides, *rel-*(1*R*,2*S*,3*R*,4*R*) *p*-menthane-1,2,3-triol 3-*O*-*β*-D-glucopyranoside (1) and *rel-* (1*S*,2*R*,3*S*) terpinolene-1,2,3-triol 3-*O*-*β*-D-glucopyranoside (2) were isolated from aqueous acetone extract of the aerial parts of *Mentha haplocalyx* Briq.. Their structures were elucidated through spectral analysis using MS and NMR spectrometers.

## Findings

**Figure 1:**
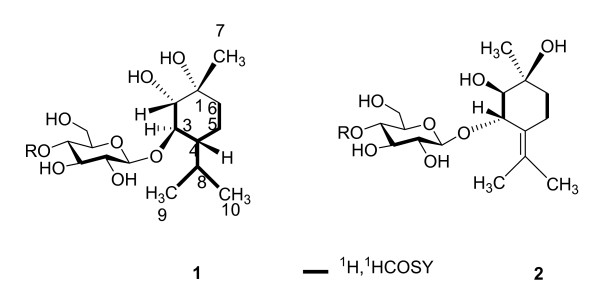
***rel-*(1*R*,2*S*,3*R*,4*R*)*p*-menthane-1,2,3-triol 3-*O*-*β*-D-glucopyranoside (1) and*rel-*(1*S*,2*R*,3*S*) terpinolene-1,2,3-triol 3-*O*-*β*-D-glucopyranoside (2) from *mentha haplocalyx* Briq**.

*Mentha* species are used for their flavoring and medicinal properties widely throughout the world [1]. *Mentha haplocalyx* Briq., is widely used in food, cosmetics and medicines, distributed in the southwest of China [2]. It has traditionally been used to treat various diseases of breath, procreation and digestive systems in China. Primary investigation on this plant has led to the isolation of polyphenolic acids, several flavonoids and monoterpenoids [3-6], and in a continuation study to obtain minor constituents, two new monocyclic monoterpenoid glycosides, *rel-* (1*R*,2*S*,3*R*,4*R*) *p*-menthane-1,2,3-triol 3-*Oβ*-D-glucopyranoside (**1**) and *rel-* (1*S*,2*R*,3*S*) terpinolene-1,2,3-triol 3-*Oβ*-D-glucopyranoside (**2**) were obtained (Figure 1). This paper deals with the isolation and identification of these new compounds.

Repeated column chromatography (CC) of the chlorophyll removal fraction in the 70% aqueous acetone extract obtained from the aerial parts of *M*. *haplocalyx* over Dianion *HP 2MG*L, MCI-gel *CHP*-*20P* and silica gel resulted in the isolation of two compounds. On the basis of spectroscopic methods, including 2D-NMR (HMQC, HMBC, ^1^H-^1^H COSY and NOESY), the structures of two new were determined as *rel-* (1*R*,2*S*,3*R*,4*R*) *p*-menthane-1,2,3-triol 3-*O*-*β*-D-glucopyranoside (1) and *rel-* (1*S*,2*R*,3*S*) terpinolene-1,2,3-triol 3-*O*-*β*-D-glucopyranoside (2).

Compound 1 was obtained as a pale amorphous powder. Its HR-ESI-MS displayed quasi-molecular-ion peak [*M* + Na]^+ ^at *m*/*z* 373.1521 ([C_16_H_30_O_8_Na]^+^), and the EI-MS gave fragment-ion peaks at *m*/*z* 171 [*M +* 1–162(glucosyl)-H_2_O]^+ ^and 153 [*M* + 1–162(glucosyl)-2H_2_O]^+ ^corresponding to a molecular formula C_16_H_30_O_8_, with the presence of 16 carbon signals in the ^13^C-NMR spectrum.

The ^1^H- and ^13^C-NMR spectral data displayed the presence of two secondary methyl *δ* 0.81 (3H, *dJ* = 7.0 Hz, H-10), 0.92 (3H, *dJ* = 7.0 Hz, H-9)], a tertiary methyl *δ* 1.21 (3H, *s*, H-7)], two methylenes *δ* 1.37 (2H, *dtJ* = 11.7, 8.3 Hz, H-5), 1.40 (1H, m, H-6α) and 1.57 (1H, m, H-6β)], four methines (two of them was oxygenated) *δ* 3.82 (1H, *dJ* = 10.8, 9.2 Hz, H-3), 3.33 (1H, *dJ* = 10.8 Hz, H-2), 2.31 (1H, m, H-8), and 1.69 (1H, m, H-4)], and an oxygentated quaternary carbon, suggesting that compound 1 was a menthane-type monoterpene with three OH-groups [7,8]. Moreover, ^1^H-^1^H COSY correlations were observed between H-C(9)/H-C(8)/H-C(10), H-C(8)/H-C(4), and H-C(6)/H-C(5)/H-C(4)/H-C(3)/H-C(2), that the deduced spin system implied that the three OH-groups were located at C(1), C(2) and C(3) in 1, respectively. In addition, one glucopyranosyl unit *δ* (H) 4.33 (1H, *dJ* = 8.2 Hz, H-(1′)), *δ*(C) 105.9 (C-1′)] was evident from ^1^H- and ^13^C-NMR of 1. The *J* value (8.2 Hz) of the anomeric proton concluded the *β*-configuration of the glucose moiety, suggesting that 1 was a *p*-menthane-1,2,3-triol glycoside. This was further confirmed by the HMBC experiment, in which correlations of the glucosyl H-1′ (*δ* 4.33) with the C(3) (*δ* 81.9) were observed. Furthermore, other HMBC correlations confirmed the structure of compound 1. Thus, these 2D-NMR methods deduced compound 1 as *p*-menthane-1,2,3-triol 2-*Oβ*-D- glucopyranoside. The coupling constants of 10.8 Hz for H-C(3)/H-C(2), 9.2 Hz for H-C(3)/H-C(4) for 1 showed that H-C(2), H-C(3) and H-C(4) were axial protons. The relative configuration at C(1) was determined from ROESY correlation of *δ* 1.21 (Me(7)) with H-2 (*δ* 3.33). It was in good agreement with those of *rel-*(1*R*,2*S*,3*R*,4*R*,6*S*) *p*-menthane-1,2,3,6-tetrol [8]. Therefore 1 should possess *rel*-(1*R*,2*S*,3*R*,4*R*)-configuration.

Compound **2** was obtained as a white amorphous powder. Its molecular formula was assigned as C_16_H_28_O_8 _on the basis of the ^13^C-NMR data and negative HR-ESI-MS (*m/z* 347.1711 [M-H]^-^), which was 2 amu less than that of 1.

The ^1^H- and ^13^C-NMR spectra of compounds 1 and 2 were very similar and gave same signals assignable to two secondary methyl, a tertiary methyl, two methylenes, two oxygenated methines, an oxygenated quaternary carbon and a *β*-D-glucopyranosyl unit. Comparison of the NMR data of 2 and 1 indicated that the only difference was the presence of two olefinic carbons in 2 instead of the two methines in 1. It suggested that a double bond situated at C(4) and C(8) positions in 2. This was supported by the IR spectrum showing a strong band at 1649 cm^-1^, probably due to a tetra-substituted double bond and the correlations of the H-C(5) with the olefinic carbons C-4 and C-8 observed in the HMBC spectrum. The ^1^H-^1^H COSY interactions of H-2 (*δ* 3.37)/H-3 (*δ* 5.02), and H-5 (*δ* 2.42, 2.14)/H-6 (*δ* 1.75, 1.33) provided C-2 and C-3 positions. This was sustained by the HMBC experiment showing correlations of H-3 (*δ* 5.02) with the C(1), C(2), C(4), C(5) and C(8) observed, respectively. It reveals a terpinolene-1,2,3-triol fragment in 2 [9]. The full assignments of the aglycon and sugar signals were carried out by HSQC, ^1^H-^1^H COSY and HMBC experiments. The HMBC correlations of glucosyl H-1′ (*δ* 4.09) in 2 with the C(3) at *δ* 76.1 confirmed the location of glucosyl at C(2). The coupling patterns [(*δ* 3.37, *dJ*_23_ = 2.86, H-2) and (*δ* 5.02, *dJ*_2, 3_ = 2.86, H-3)] demonstrated that H-C(2) and H-C(3) were axial-equatorial or equatorial-equatorial couplings. In the ROESY experiments that H-2 and Me-7 ROESY correlation was missing, while H-3 and Me-7 was present. It was illustrated that H-2 and H-3 *trans* located on the alpha and beta face, respectively. Therefore, the structure of 2 was determined to be *rel-* (1*S*, 2*R*, 3*S*) terpinolene-1,2,3-triol 3-*Oβ*-D- glucopyranoside [9].

## Additional material

### Experimental part

#### Genaral

Optical rotations were measured on a P-1020 Polarimeter (JASCO, Tokyo, Japan). IR spectra: *IR-450* spectrometer with KBr pellets; ^1^H- and ^13^C-NMR, HSQC, HMBC and ^1^H-^1^H COSY, ROESY spectra: *DRX-500* spectrometers operating at 500 MHz for ^1^H, and 125 MHz for ^13^C, respectively, in CD_3_OD; ESI-MS, EI-MS and HR-EI-MS: *APEX II FT-ICR* and VG-ZAB-HS spectrometer. Column chromatography (CC): Dianion *HP 2MG*L, Silica gel, and MCI-gel *CHP 20P*. TLC: silica gel *G* plates with CHCl_3_-MeOH-H_2_O (8:2:0.2 or 7:3:0.5).

#### Plant material

The aerial parts of *M*. *haplocalyx* was purchased from Beijing TongRenTang Medicinal Material Co., Beijing, China, in June 2006, and identified by Prof. *B*. *L.*, in Beijing University of Chinese Medicine.

#### Extraction and isolation

The aerial parts of *M*. *haplocalyx* (5.0 kg) was extracted with 70% aqueous acetone three times (10 L × 3) at room temperature. After removal of the organic solvent under reduced pressure, the aqueous solution was partitioned with ethyl ether to yield ethyl ether and aqueous fraction. The aqueous fraction was concentrated to a small volume (200 ml) and subjected to a Dianion *HP 2MG*L column, eluting with H_2_O-MeOH (1:0–0:1) to afford six fractions (*Frs*. 1–6). *Frs*. 4 (4 g) was subjected to CC on silica gel (CHCl_3_/MeOH, 9:1–7:3) and MCI-gel *CHP20P* eluted with H_2_O/MeOH to give 1 (3 mg) and **2** (4 mg).

(*1*R*,2*S*,3*R*,4*R) *p-menthane-1,2,3-triol* 3-*O*-*β*-D*- glucopyranoside* (1): pale amorphous powder, [α]${}_{\text{D}}^{20}$ = +5.1° (*c* = 0.187, MeOH), IR (KBr): 3363, 2919, 1372, 1259, 1162, 1034. ^1^H-NMR (CD_3_OD, 500 MHz) and ^13^C-NMR (CD_3_OD, 125 MHz): Table 1 showed. EI-MS *m/z* 171 [*M* + 1 − 162(glucosyl)-H_2_O] ^+ ^and 153 [*M* + 1 − 162(glucosyl)-2H_2_O] ^+^, 135, 127, 112, 97, 84, 73, 55. HR-ESI-MS: *m/z* 373.1521 ([C_16_H_30_O_8_Na]^+^), calcd for C_16_H_30_O_8_Na, 373.1990.

**Table 1:** ^***13***^***C-*(125 MHz)*and***^***1***^ ***H-*(500 MHz)*NMR spectroscopic data for*1–2 in CD**_**3**_**OD (*δ*in ppm,*J*in Hz)**

**No.**	**1**		**2**	
	***δ***_C_	***δ***_H_	***δ***_C_	***δ***_H_
1	73.8	74.6		
2	77.9	3.33 (1H, d, *J* = 10.8 Hz)	79.1	3.37 (d, *J* = 2.86 Hz)
3	81.9	3.82 (1H, dd, *J* = 10.8, 9.2 Hz)	76.1	5.02 (d, *J* = 2.86 Hz)
4	41.7	1.69 (1H, m)	129.1	
5	18.8	1.37 (2 H, dt, *J* = 11.7, 8.3 Hz)	23.5	2.14 (1 H, m, H-5a) 2.42 (1 H, m, H-5β)
6	33.2	1.40 (1 H, m, H-6a) 1.57 (1 H, m, H-6β)	40.1	1.33 (1 H, m, H-6a) 1.75 (1 H, m, H-6β)
7	28.1	1.21 (3 H, s)	22.1	1.36 (3 H, s)
8	25.6	2.31 (1 H, m)	131.7	
9	21.6	0.92 (3 H, d, *J* = 7.0 Hz)	20.3	1.78 (3 H, s)
10	16.2	0.81 (3 H, d, *J* = 7.0 Hz),	20.6	1.74 (3 H, s)
Glc-1′	105.9	4.33 (1 H, d, *J* = 8.2 Hz)	99.7	4.09 (1 H, d, *J* = 8.2 Hz)
2′	75.3	3.29 (1 H, m)	74.6	3.29 (1 H, m)
3′	77.9	3.20 (1 H, m)	78.5	3.54 (1 H, m)
4′	71.3	3.33 (1 H, d, *J* = 9.2 Hz)	71.5	3.13 (1 H, m)
5′	76.8	3.79 (1 H, m)	77.8	3.40 (1 H, m)
6′	62.6	3.88 (dd, 9.8, 2.0) 3.55 (dd, 12.0, 6.0)	62.6	3.82 (dd, 12.1, 2.3) 3.67 (dd, 12.1, 6.4)

*(1*S*,2*R*,3*R*) terpinolene −1,2,3-triol* 3-*O*-*β*-D*- glucopyranoside* (**2**): pale amorphous powder, [α]${}_{\text{D}}^{20}$= –44.9° (*c* = 0.323, MeOH), IR (KBr): 3308, 2938, 1649, 1449, 1341, 1259, 1076. ^1^H-NMR (CD_3_OD, 500 MHz) and ^13^C-NMR (CD_3_OD, 125 MHz): Table 1 showed. ESI-MS: *m/z* 347.2 [*M*-H]^-^, 185.1 [*M*-H-162(glucosyl)]^-^. HR-ESI-MS: *m/z* 347.1711 [*M*-H]^-^, calcd for C_16_H_27_O_8_, 347.1710.

## Competing interests

The authors declare that they have no competing interests.

## Authors’ contributions

GS carried out the chemical analysis-structure elucidation and drafted the Manuscript; CX carried out the chemical and biological studies; BL conceived of the study and its design and coordination of the scientific teams. All authors have read and approved the final manuscript.
